# Case Report: An extremely rare occurrence of recurrent inguinal low-grade fibromyxoid sarcoma involving the scrotum

**DOI:** 10.12688/f1000research.24287.2

**Published:** 2020-10-22

**Authors:** Samy Chitayat, Rodrigo Barros, José Genilson Ribeiro, Heleno Augusto Moreira Silva, Flávio Rondinelli Sá, Bruno de Souza Bianch Reis, Angelo Maurilio Fosse Junior

**Affiliations:** 1 Urology, Fluminense Federal University, Niterói, Rio de Janeiro, 24220900, Brazil; 2 Pathology, Fluminense Federal University, Niterói, Rio de Janeiro, 26299000, Brazil

**Keywords:** Low-grade fibromyxoid sarcoma, sarcoma, scrotum sarcoma

## Abstract

Low-grade fibromyxoid sarcoma (LGFMS) is a rare sarcoma subtype. The most common tumor locations are the deep soft tissue of extremities or trunks. We report a rare case of recurrent LGFMS in the inguinal region involving the scrotum and both testicles. A 38-year-old male patient reported a history of multiple nodular lesions in the left inguinal region accompanied by local inflammation. The patient was submitted for local resection of the lesion at our institution, with histopathological diagnosis of LGFMS. He missed his follow-up, returning with a large bulge in the left inguinal region involving the scrotum with signs of tissue necrosis and local purulent discharge. Surgical exploration was performed and the patient underwent tumor resection in the left inguinal region and the entire scrotum, with bilateral orchiectomy, with the margins enlarged to the right inguinal region and proximal surface of the penis. Local reconstruction was performed with a left fascia lata tensor muscle flap and ipsilateral thigh coverage using partial skin graft. On microscopic examination, the tumor showed spindle cells arranged in bundles, with abundant collagen and myxoid stroma with interspersed prominent vessels. The immunohistochemical study carried out showed immunoreactivity with Ki67 (<5%), immunonegativity with desmin and S100, confirming the diagnosis of LGFMS. Postoperative recovery was good and no recurrence was seen after two years. The patient is in good health, realizing multidisciplinary outpatient follow-up and performing continuous testosterone replacement. Surgical resection with negative margins for localized disease remains the standard treatment for LGFMS.

## Introduction

Low-grade fibromyxoid sarcoma (LGFMS) is a rare sarcoma subtype, first described by Evans in 1987 ^1^. The most common tumor locations are the deep soft tissue of extremities or trunks ^2^. Paratesticular LGFMS are rare with few cases published in the literature ^3, 4^. The etiology still unknown and the incidence is 0.18 per million, representing 0.6% of all soft tissue sarcomas ^5^.

Microscopy reveals bland spindle cell tumors with angulated nuclei, scant cytoplasm arranged in a whorled pattern with cells that are frequently immunoreactive to mucin 4 ^2^. Despite its deceptively indolent clinical behavior and benign histological appearance, LGFMS has a high tendency for local recurrence and late distant metastasis ^6^. The current treatment includes surgical excision with clear margins for localized disease with or without radiotherapy, while conventional systemic therapy has limited efficacy in advanced LGFMS ^7^.

Here, we report a rare case of recurrent LGFMS in the inguinal region involving the scrotum and both testicles. To the best of our knowledge, there is no case described with this rare presentation.

## Case presentation

### Patient information and medical history

**Figure 1.**
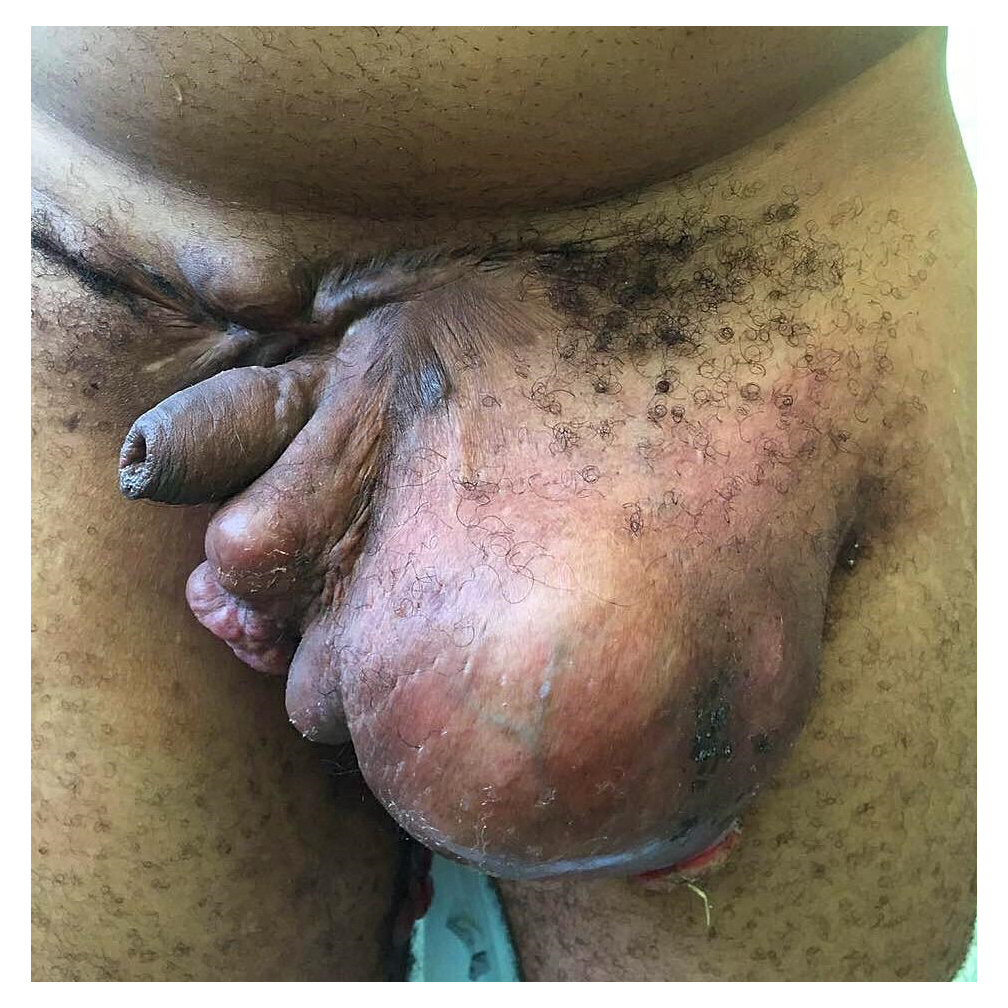
Recurrent inguinal low grade fibromyxoid sarcoma involving scrotum in a 38-year-old patient.

A 38-year-old male patient reported a history of multiple nodular lesions in the left inguinal region accompanied by a local inflammatory process since the age of 13. Since then, he had undergone multiple surgical procedures performed by different health services, with clinical and histopathological diagnosis of complicated hidradenitis. The patient was submitted to local resection of the lesion at our institution, with histopathological diagnosis of LGFMS. He missed his follow-up in 2009, only returning in 2017 with a large bulge in the left inguinal region, bigger than primary tumor, involving the scrotum with signs of tissue necrosis and local purulent discharge ( Figure 1).

### Diagnosis and intervention

**Figure 2.**
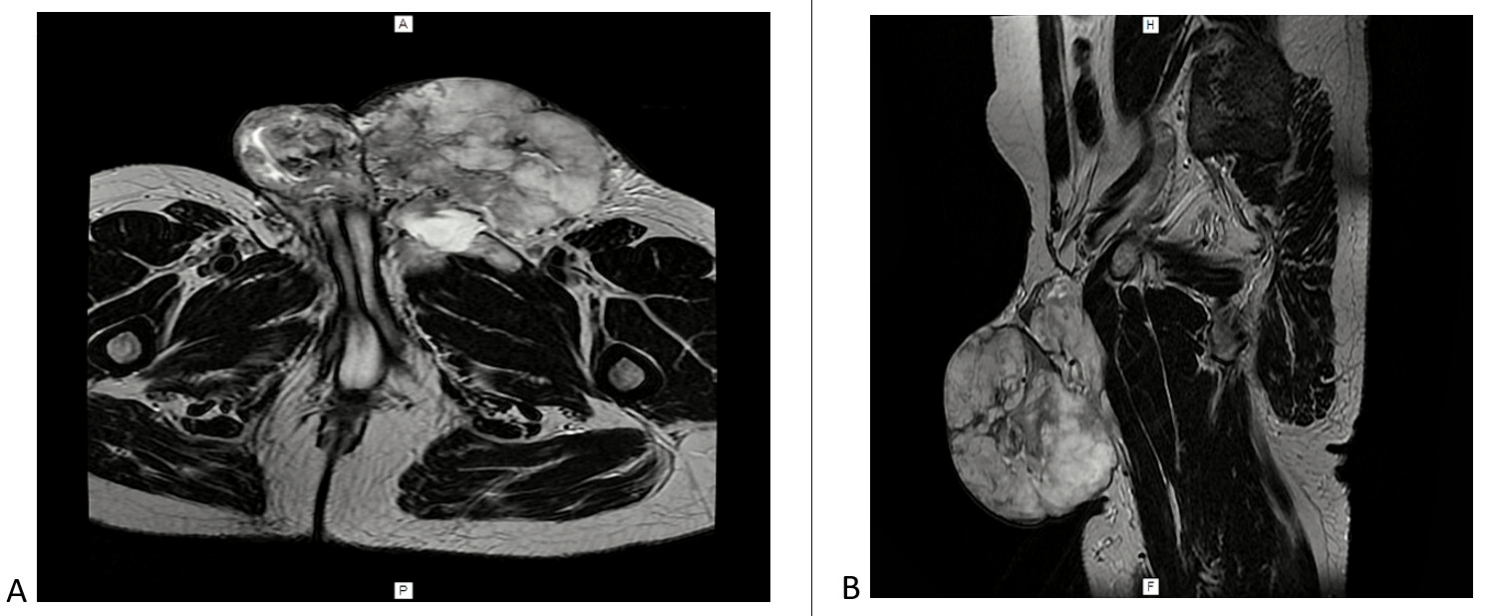
Magnetic resonance imaging. **A**) Cross section revealing inseparable inguinal mass with structures of the scrotum. **B**) Sagittal section showing the relationship between the tumor mass and the adductor musculature of the left thigh.

Magnetic resonance imaging (MRI) showed a mass of lobulated contours and partially defined limits, measuring 13 cm in the largest diameter, located in the left scrotum and extending to the perineal region and the medial aspect of the thigh, with invasion of the ipsilateral adductor muscles, not separable from the left testicle ( Figure 2A and 2B).

**Figure 3.**
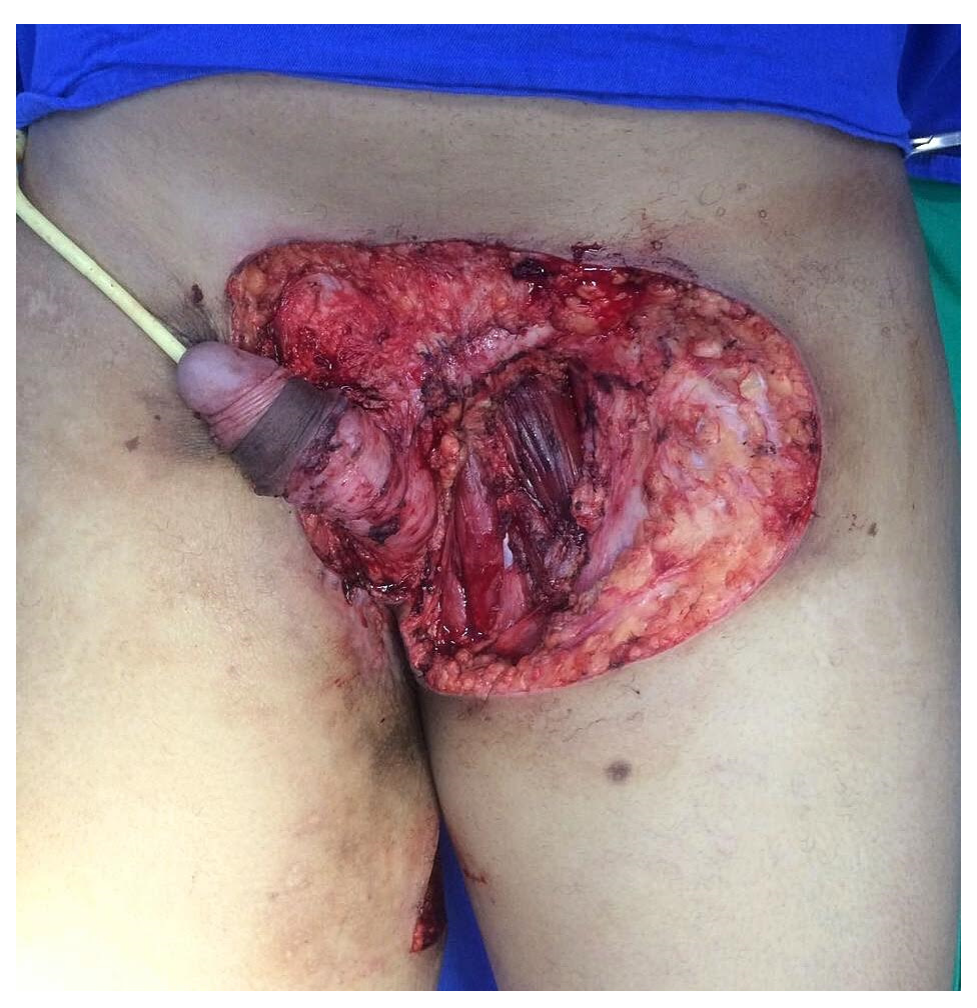
Aspect after resection of inguinal low grade fibromyxoid sarcoma involving the scrotum.

**Figure 4.**
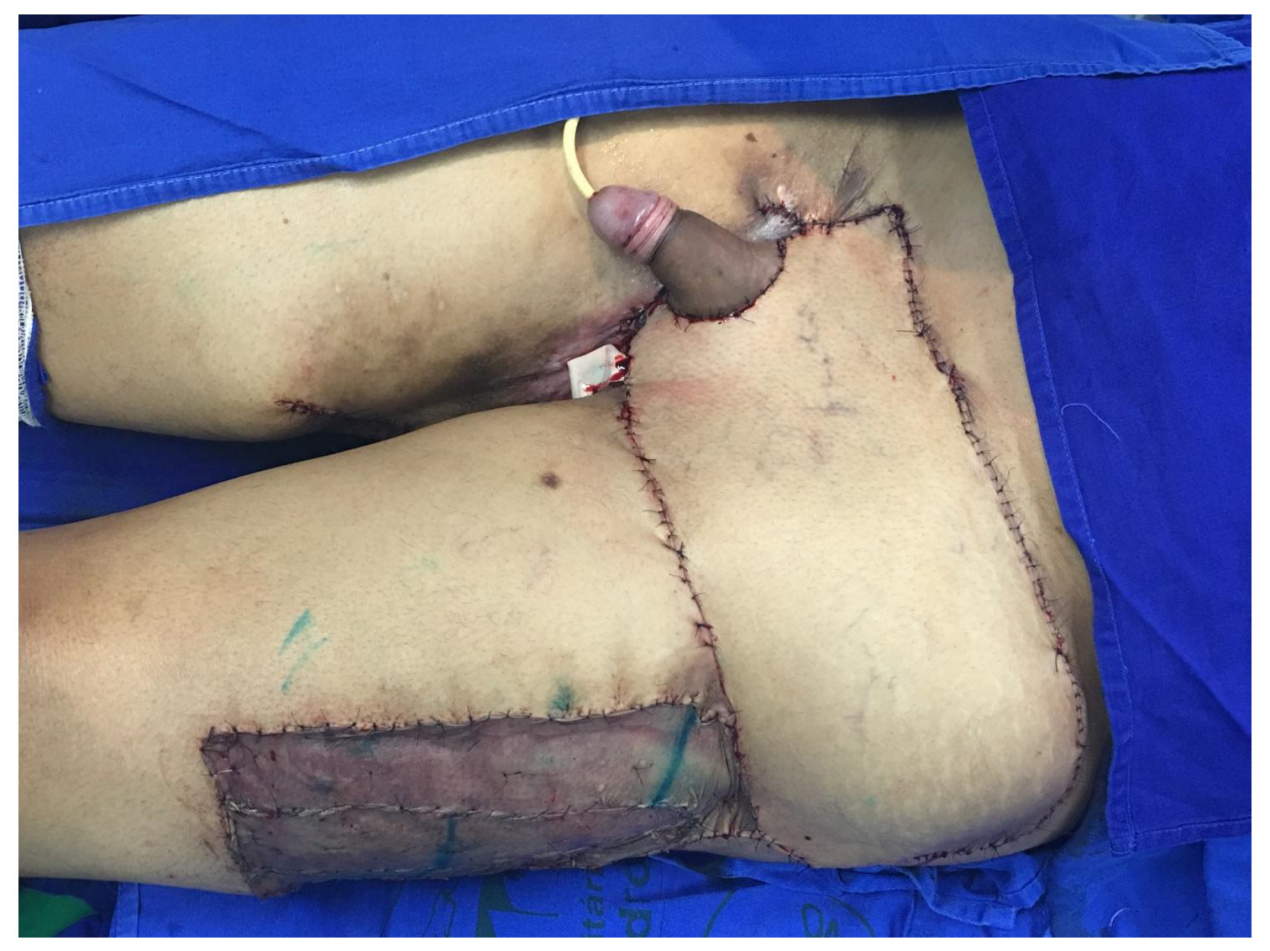
Aspect after reconstruction.

Given this case of a large recurrent LGFMS, the patient was scheduled for surgical intervention. Under general anesthesia, the patient was placed in supine position and the intraoperative findings were compatible with the MRI results, additionally revealing the involvement of the right testicle. The patient underwent tumor resection in the left inguinal region and the entire scrotum, with bilateral orchiectomy, with the margins enlarged to the right inguinal region and proximal surface of the penis, this stage of the surgery being performed by the urology team ( Figure 3). Local reconstruction was performed by the plastic surgery team, with a left fascia lata tensor muscle flap and ipsilateral thigh coverage using partial skin graft ( Figure 4).

**Figure 5.**
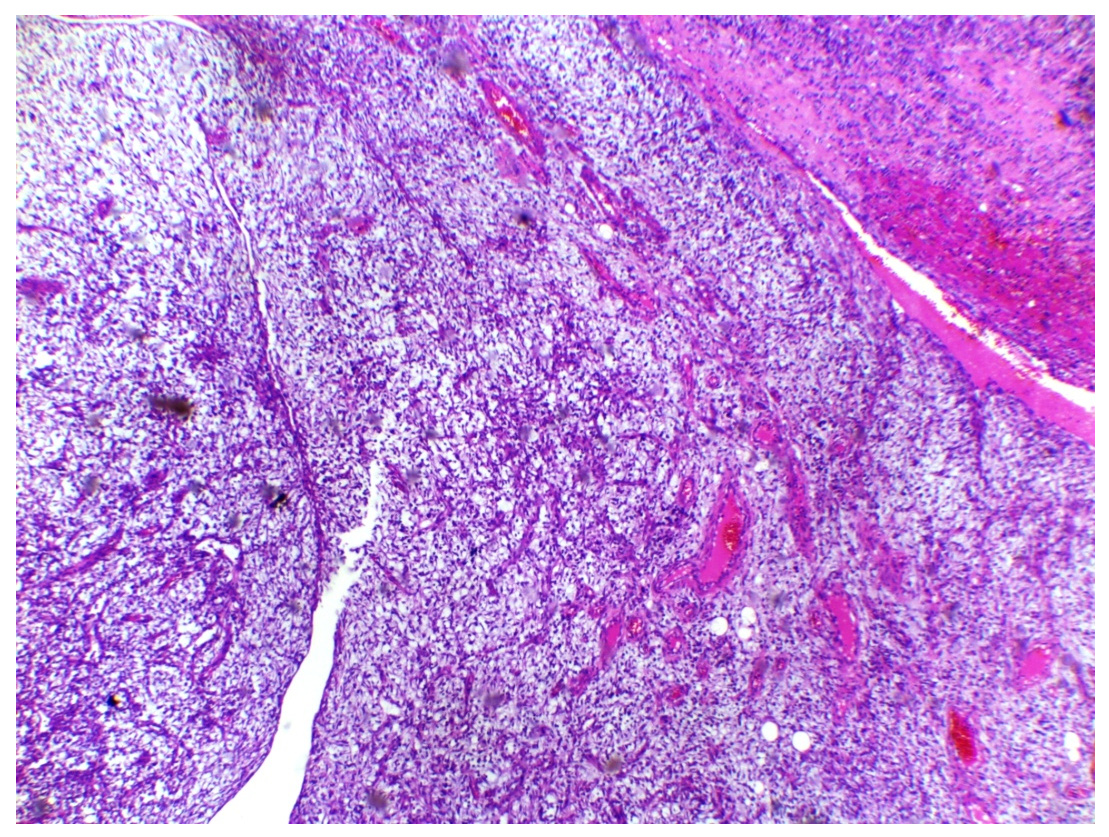
Histological image of the low grade fibromyxoid sarcoma showing an admixture of collagenized and hypocellular myxoid zones with arcades of small vessels.

On microscopic examination, the tumor showed an admixture of hypocellular zone and more cellular, spindle cell nodule. Arcades of small vessels with perivascular sclerosis were seen ( Figure 5). The immunohistochemical study carried out showed immunoreactivity with Ki67 (<5%), immunonegativity with desmin and S100, confirming the diagnosis of grade 2 LGFMS according to American College of Pathology staging.

### Follow-up

Postoperative recovery was good and no recurrence was seen after two years. The patient is in good health, realizing multidisciplinary outpatient follow-up at least every six months in the departments of oncology, urology, plastic surgery and endocrinology, where interviews, physical examination, image exams and testosterone replacement therapy control are carried out. MRI of the abdomen and pelvis, in addition to chest CT are performed every six months. Testosterone replacement is being performed continuously with intramuscular injections of 1,000 mg of testosterone undecanoate once every 12 weeks, keeping testosterone levels in the reference range without side effects.

## Discussion

LGFMS is a recently recognized soft tissue tumor that was first reported in 1987 by Evans as a metastasizing tumor with a deceptively benign histological appearance, affecting predominantly adults during the fourth decade of life ^1^. Patients are often misdiagnosed with fibromatosis, neurofibroma or other benign conditions instead of LGFMS ^8^. In our case, the patient underwent multiple procedures without success due to misdiagnosis of hidradenitis. LGFMS generally occurs in the lower proximal extremities and trunk, but is also infrequently described as arising from the inguinal region and the chest wall ^9^. Despite its relatively low-grade histology, local postsurgical recurrence and metastases to lungs and bone are frequently seen and can appear several years after primary surgery ^10^. We present a case in which a primary tumor of the inguinal region relapsed after multiple surgical treatments and aggressively invaded adjacent structures, including both testicles.

Radiological examination has an important role in the diagnosis of LGFMS. However, it is difficult to distinguish LGFMS from other mesenchymal tumors due to the rarity. CT scan and MRI can identify the lesions and help evaluate relationships with adjacent structures. In imageological examination, LGFMS is generally seen as a solitary and well circumscribed lesion, although it can be present as multiple infiltrating masses upon recurrence ^11^. MRI techniques can be more helpful than CT to detect the fibrous and myxoid components of the tumor according to the T1/T2 signal intensity ^12^. In our case, MRI was essential for surgical programming, presenting findings similar to those during surgery.

The definite diagnosis depends on histopathological examination. Histologically, the tumor has a deceptively benign appearance, making diagnosis a challenge. The immunohistochemistry can exclude entities in differential diagnosis and the diagnostic marker for LGFMS is MUC4. However, this marker not available in the lab to perform on this case. The main differential diagnoses are Fibromatosis, Fibrosarcoma, Myxofibrosarcoma, Myxoid neurofibroma, Nodular fasciitis, Myxoid dermatofibrosarcoma, Malignant peripheral nerve sheath tumor. Therefore, it is essential for the diagnosis to be confirmed by an expert soft-tissue pathologist ^13^. The patient in our study had undergone multiple surgical resections without success, due to mistaken histopathological diagnosis of hidradenitis, before being examined at our institution.

Surgical resection with negative margins for localized disease remains the standard treatment for LGFMS. However, treatments for advanced disease are limited. Radiotherapy has questionable efficacy, being reserved for cases of positive margins, recurrence or metastasis. Chemotherapy is usually reserved for patients with metastatic disease. However, there are no data to support the use of any systemic or locoregional treatments ^5^. Chamberlain *et al.* recently described their experience with non-surgical therapies to treat LGFMS. According to the authors, systemic therapy has limited efficacy in advanced LGFMS ^7^. Unlu *et al.*, reported two cases of paratesticular LGFMS treated with simple orchiectomy. The patients had residual mass but did not accept additional treatment and both died of the disease, emphasizing the importance of radical surgical treatments ^4^. Despite tumor recurrence in our case, the patient did not present metastasis after aggressive surgical treatment and it was not necessary to perform adjuvant treatment.

To the best of our knowledge, there is no case described with recurrent LGFMS in the inguinal region involving the scrotum and both testicles. The patient was properly treated through tumor resection and local reconstruction. However, this study has limitations due to the short follow-up period.

## Conclusion

LGFMS is a rare sarcoma subtype but one which should be considered in nodular lesions in the inguinal region. Histologically, the tumor has a deceptively benign appearance, making diagnosis a challenge. If missed, adjacent structures such as the scrotum can be aggressively involved. Surgical resection with negative margins for localized disease remains the standard treatment.

## Data availability

All data underlying the results are available as part of the article and no additional source data are required.

## Consent

Written informed consent for publication of their clinical details and clinical images was obtained from the patient.
