# Supplementary material for: Case Report: An extremely rare occurrence of recurrent inguinal low-grade fibromyxoid sarcoma involving the scrotum
Source: F1000Res. 2020 Oct 22;9:789. Originally published 2020 Jul 29. [Version 2] doi: 10.12688/f1000research.24287.2 (PMC7596807; doi:10.12688/f1000research.24287.2)
Supplement: Peer review history

# Peer review: Reviewer response for version 2

**DOI:** 10.5256/f1000research.30315.r73563

**Published:** 2020-10-29

**Authors:** Nasir Ud Din

**Affiliations:** 1 Department of Pathology and Laboratory Medicine, Aga Khan University Hospital, Karachi, Pakistan

Queries have been addressed. Recommend for indexing.

Are enough details provided of any physical examination and diagnostic tests, treatment given and outcomes?

Yes

Is the case presented with sufficient detail to be useful for other practitioners?

Partly

Is sufficient discussion included of the importance of the findings and their relevance to future understanding of disease processes, diagnosis or treatment?

Yes

Is the background of the case’s history and progression described in sufficient detail?

Partly

Reviewer Expertise:

I am a histopathologist with expertise in soft tissue and bone sarcomas.

I confirm that I have read this submission and believe that I have an appropriate level of expertise to confirm that it is of an acceptable scientific standard.

# Peer review: Reviewer response for version 1

**DOI:** 10.5256/f1000research.26796.r72099

**Published:** 2020-10-12

**Authors:** Nasir Ud Din

**Affiliations:** 1 Department of Pathology and Laboratory Medicine, Aga Khan University Hospital, Karachi, Pakistan

- The authors have reported a case of recurrent scrotal LFMS. The initial and recurrent tumor size needs to be reported. Was recurrent tumor bigger than primary tumor?
- The authors claim their case to be first case in this location. However, a previous publication (Unlü *et al.*, 2015 ^1^) has reported two paratesticular LGFMS. Please read that article and cite.
- Apart from morphology, the diagnostic marker for LGFMS is MUC4. Was this marker not available in the lab to perform on this case?
- In the pathology section, a differential diagnoses should be first described followed by mentioning appropriate IHC.

Are enough details provided of any physical examination and diagnostic tests, treatment given and outcomes?

Yes

Is the case presented with sufficient detail to be useful for other practitioners?

Partly

Is sufficient discussion included of the importance of the findings and their relevance to future understanding of disease processes, diagnosis or treatment?

Yes

Is the background of the case’s history and progression described in sufficient detail?

Partly

Reviewer Expertise:

I am a histopathologist with expertise in soft tissue and bone sarcomas.

I confirm that I have read this submission and believe that I have an appropriate level of expertise to confirm that it is of an acceptable scientific standard, however I have significant reservations, as outlined above.

## References

[1] : Paratesticular sarcomas: A report of seven cases. *Oncol Lett*.2015;9(1) : 10.3892/ol.2014.2629 308-312 10.3892/ol.2014.2629 25435981PMC4246635

# Peer review: Reviewer response for version 1

**DOI:** 10.5256/f1000research.26796.r68357

**Published:** 2020-08-19

**Authors:** Felipe Lott

**Affiliations:** 1 Department of Urology, Instituto Nacional de Câncer (INCA), Rio de Janeiro, Brazil

The case is well described, with good quality photos and image exam. A well documented pathology analysis was made. It is a rare pathology and this article brings more substance for future diagnosis alert. The treatment is correctly described and clear. All case history is acceptable. I think there is a reason for the article to be indexed without necessity for revision.

Are enough details provided of any physical examination and diagnostic tests, treatment given and outcomes?

Yes

Is the case presented with sufficient detail to be useful for other practitioners?

Yes

Is sufficient discussion included of the importance of the findings and their relevance to future understanding of disease processes, diagnosis or treatment?

Yes

Is the background of the case’s history and progression described in sufficient detail?

Yes

Reviewer Expertise:

I am an expert in urology oncology at Brazilain National Cancer Institute.

I confirm that I have read this submission and believe that I have an appropriate level of expertise to confirm that it is of an acceptable scientific standard.
